# Physician-Modified Endografts for Repair of Complex Abdominal Aortic Aneurysms: Clinical Perspectives and Medico-Legal Profiles

**DOI:** 10.3390/jpm14070759

**Published:** 2024-07-17

**Authors:** Giovanna Ricci, Filippo Gibelli, Ascanio Sirignano, Maurizio Taurino, Pasqualino Sirignano

**Affiliations:** 1 Section of Legal Medicine, School of Law, University of Camerino, 62032 Camerino, Italy; giovanna.ricci@unicam.it (G.R.); ascanio.sirignano@unicam.it (A.S.); 2 Vascular and Endovascular Surgery Unit, Sant’Andrea Hospital of Rome, Department of Molecular and Clinical Medicine, “Sapienza” University of Rome, 00185 Rome, Italy; maurizio.taurino@uniroma1.it; 3 Vascular and Endovascular Surgery Unit, Sant’Andrea Hospital of Rome, Department of General and Specialistic Surgery, “Sapienza” University of Rome, 00185 Rome, Italy; pasqualino.sirignano@uniroma1.it

**Keywords:** abdominal aortic aneurysm, EVAR, hostile anatomy, EVAR outcomes, IFU, instruction for use, physician modified endograft (PMEG), ethical considerations, informed consent

## Abstract

Standard endovascular aortic repair (EVAR) has become the standard of care for treating infrarenal abdominal aortic aneurysms (AAAs) in patients with favorable anatomies, while patients with challenging AAA anatomies, and those with suprarenal or thoraco-abdominal aneurysms, still need alternative, more complex, solutions, including custom-made branched or fenestrated grafts, which are constrained by production delay and costs. To address urgent needs and complex cases, physicians have proposed modifying standard endografts by manually creating graft fenestrations. This allows for effective aneurysm exclusion and satisfactory patency of visceral vessels. Although physician-modified grafts (PMEGs) have demonstrated high technical success, standardized creation processes and long-term safety data are still lacking, necessitating further study to validate their clinical and legal standing. The aim of this article is to illustrate the state of the art with regard to this surgical technique, summarizing its origin, evolution, and the main clinical evidence supporting its effectiveness. The paper also aims to discuss the main medico-legal issues related to the use of PMEGs, with particular reference to the issue of safety related to the standardization of the surgical technique, medical liability profiles, and informed consent.

## 1. Introduction

In the last three decades, endovascular aortic repair (EVAR) has emerged as a safe and valid option for the treatment of abdominal aortic aneurysms (AAAs), and nowadays, EVAR could be considered as the first-line treatment for most patients with feasible anatomy [1,2,3,4,5]. Nevertheless, all the available guidelines suggest refraining from using standard endografts in challenging aortic neck anatomies and the adoption of different solutions to achieve and maintain an effective AAA exclusion [4,5,6]. 

Recently, different custom-manufactured branched/fenestrated (B/FEVAR) grafts are become widely available, except in the USA, where there is only one Food and Drug Administration (FDA)-approved fenestrated endograft (Zenith Fenestrated Endovascular Graft; Cook Medical, Bloomington, IN, USA) to treat AAA defined as “complex” in the latest version of the European guidelines [5]. However, the applicability of custom-made B/FEVAR endografts is still severely limited by the waiting time for manufacturing (from 4 to 6 weeks), highly restrictive anatomic standards, and expensive procedure-related costs [7,8].

Unfortunately, the latter is not acceptable for patients at high surgical risk in association with urgent/emergent situations. To fulfill these therapeutic limitations, physicians have developed different techniques to create fenestration in standard devices (physician modified endograft—PMEG) and to elude the restrictions raised by custom-made B/FEVAR endografts. Despite encouraging early results published in the literature [9,10,11,12], PMEGs lack standardization in their creation process, as well as comprehensive data on long-term durability and patient safety. Therefore, the aim of the present study is to provide an overview of current evidence regarding PMEG creation techniques, present available results, and assess their clinical value in light of medico-legal considerations.

Table 1 presents a brief comparison of the characteristics of traditional and customized vascular endoprostheses of the abdominal aorta. 

As far as costs are concerned, a very recent study highlights the significant financial burden associated with PMEGs in the treatment of thoracoabdominal aortic aneurysms (TAAAs) compared to commercially available fenestrated-branched devices and traditional open surgical repair (OSR). The retrospective analysis, covering procedures carried out at an academic medical center from January 2018 to December 2022, revealed that PMEG repairs incurred a total contribution margin (CM) of USD 110,000, starkly contrasting with the USD 18,000 CM for the Cook Zenith-Fenestrated (ZFEN) graft and USD 294,000 for OSR. The primary cost driver for PMEGs was the device cost, which was almost double that of ZFEN (USD 46,000 vs. USD 25,000, *p* < 0.05), with the Extent II TAAA repairs being the most expensive, at USD 59,000 per case. To achieve financial viability, the study suggests a need for reducing device costs or increasing reimbursement rates by approximately USD 9000 for PMEG procedures [13].

## 2. PMEG Creation Techniques

**Figure 1:**
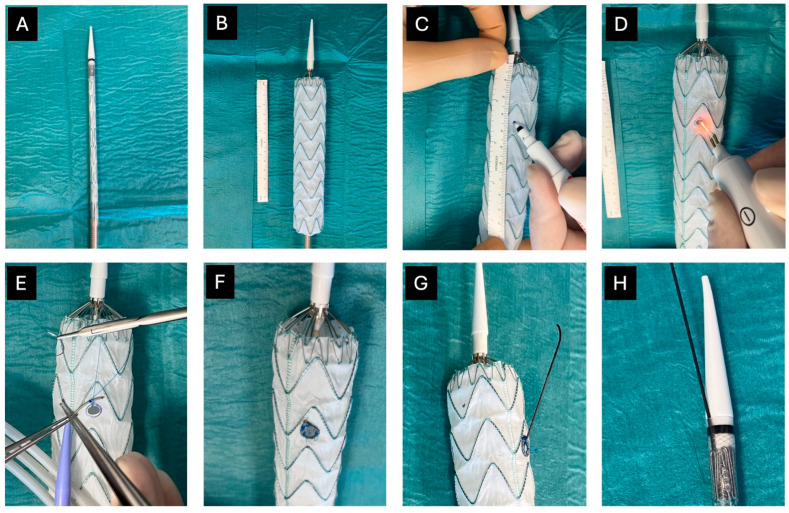
PMEG creation technique: (**A**) standard available devices; (**B**) unsheathed device; (**C**) fenestration site identification; (**D**) fenestration creation by electrocautery; (**E**) fenestration reinforcing with radiopaque wire tip; (**F**) fenestration in its final configuration; (**G**) precannulated wire (optional); (**H**) resheathed graft.

Albeit in the absence of an industrialized process for their creation, fenestrated PMEGs have been made essentially in the same manner by all the involved operators worldwide since the first report by Uflacker et al. [14]: starting from commercially available aortic endografts and endovascular ancillary devices, after careful preoperative planning with dedicated software, the aortic device to be modified is unsheathed on the back table, and heat electrocautery is used to create fenestrations in it. Then, each fenestration is reinforced with a nitinol wire and prolene suture ring. After the fenestrations are constructed, the graft is resheathed into its original delivery sheath (Figure 1).

Oderich and Ricotta [15] suggest unsheathing only the area of the device that should be modified if a single proximal fenestration is need or, alternatively, to unsheathe the entire device if multiple fenestrations should be performed. The authors also suggest using the Zenith endograft (Cook Medical, Bloomington, IN, USA), which could be relatively easy resheathed using the peel-away sheath that is provided by the manufacturer and located across the valve. The peel-away sheath facilitates the resheathing process by allowing the controlled, step-by-step re-encapsulation of the graft. By peeling back the sheath in sections, it ensures the precise alignment and protection of the graft, minimizing the risk of damage or contamination. Each fenestration should be carefully premarked in the main stent graft according to the preoperative planning. It is created via an ophthalmologic cautery with fine, meticulous, and delicate movements to avoid creating an excessively large fenestration. Thereafter, fenestration is reinforced with a gold nitinol wire fixed by a suture with 5/0 prolene. The authors also suggest creating fenestrations round in shape and located away from the stent struts. To improve PMEG orientation during deployment, a longitudinal anterior marker is placed at the 12 o’clock position and a transverse posterior marker at the 6 o’clock position in their experience. Lastly, a constraining wire is used with the PMEG graft to allow the rotational and axial movement of the stent during its deployment.

The PMEG construction technique, as described, has remained essentially unchanged over the years with a great majority of authors suggesting using bifurcated or tubular devices by Cook Medical [16,17,18,19] or the Valiant (Medtronic AVE, Santa Rosa, CA, USA) thoracic stent graft in selected cases [20]. More recently, Pyun and Han, in a very elegant review on PMEG, reported their technique as performed in a physician-sponsored IDE protocol (G200159) [9]. In their experience, the Zenith Alpha Thoracic or TX2 (Cook Medical) proximal component thoracic stent-graft is unsheathed, and one of the trigger wires is retrieved from the inner cannula of the delivery system to function as a diameter-limiting wire. Then, the wire is rerouted posteriorly through each stent along the posterior wall. Fenestrations are then created using an ophthalmic cautery (8 mm fenestrations for the visceral vessels and 6 mm for the renal arteries). Each fenestration is reinforced with a double layer of the Amplatz Goose Neck Snare (Medtronic), which is secured around each fenestration with 6-0 Gore-Tex (W.L. Gore & Associates; Flagstaff, AZ, USA) locking sutures. Consistently with previous reported experiences, these Authors suggest radiopaque markers can be sutured to differentiate the fenestration, especially when target vessels are near each other. Varying degrees of temporary diameter-constraining ties can be added to a trigger wire rerouted along the posterior aspect of the stent graft to preserve the working space for target vessel catheterization. Lastly, the device is reintroduced into its delivery sheath with the assistance of multiple temporary silk sutures.

Although most of the published experiences were performed using Cook devices, Ducasse recently reported the feasibility of the PMEG with the TREO bifurcated graft (Terumo Aortic, Sunrise, FL, USA), finding no significant differences from previously described techniques [21]. 

A very promising frontier that deserves further development is 3D model-assisted planning (3DMA). A recent study examined 3D model-assisted planning for the fenestration design of 32 PMEGs used in the treatment of complex aortic aneurysms. Multiple differences emerged between the manual and 3D-assisted planning measurements, but they were clinically irrelevant, as neither the rate of branch preservation nor the complication rate changed during the patients’ observation period of just over 1 year (no patients experienced complications) [22].

## 3. Clinical Data

Year by year, the treatment experiences of complex AAAs, as well as those of thoraco-abdominal aortic aneurysms (TAAAs), are continuously increasing, with clinical results that are becoming increasingly encouraging. 

Oderich and colleagues reported a series of 30 patients (47% with TAAA) in whom 85 fenestrations were performed, with a success rate of 98%, and one perioperative death. At a median follow-up of 14 months, the target vessels patency rate was 97%, and the freedom from endoleak rate was 88% [15]. 

In 2012, Starnes and colleagues [17] described their initial experience on 47 consecutive patients with juxtarenal AAA treated with PMEGs, accounting for 82 fenestrations; the technical success rate was 98%, and the perioperative mortality rate was 2%. Since then, in an updated publication on midterm results (64 consecutive patients and 145 total fenestrations), they reported a 30-day mortality rate of 5.1%, with 100% of cases showing no stent-graft displacement and no instances of rupture or the need for open surgical repair within 12 months. Two patients presented a high-flow endoleak at 12 months. Sac stability or shrinkage was seen in 97.7% of patients at 1 year and 95.2% of patients at 3 years [18]. 

A recent report from a high-volume aortic center in Hamburg [20] demonstrated similarly high technical success of PMEGs, even in the setting of contained rupture. Their group of 21 patients included 11 with TAAAs and 13 individuals with contained ruptures. Technical success of PMEG implantation was accomplished in all patients. Thirty-day survival was 95%. Two patients experienced permanent paralysis as a result of intraoperative hypotension caused by rupture. One patient experienced a late aneurysm-related death after developing an aortoenteric fistula. At 11.2 months’ follow-up, visceral target vessels were all patent. Interestingly, Authors suggested that the PMEG technique is useful even in European centers, where the off-the-shelf devices are available on the market, because the surgeon-modified stent-graft facilitate a prompt and anatomically correct reconstruction for those patients unsuitable for off-the-shelf standard devices. 

Similarly, Georgiadis and colleagues [23] published a systematic review comparing off-the-shelf grafts with PMEGs in 308 patients with complex AAAs. One-third of the evaluated patients were emergently treated. Major adverse events occurred in 12.8% of the PMEGs and 7.4% of off-the-shelf graft patients; death occurred in 3.2% of the PMEG group. Regarding clinical success, graft patency, and mortality, the authors concluded that both techniques were effective and safe, yielding similar results. 

O’Donnell and colleagues [24] analyzed the outcomes of 1396 complex EVARs using the national Vascular Quality Initiatives database: 880 fenestrated grafts using the commercially available ZFEN (Cook Medical), 256 PMEGs, and 260 chimney/snorkel EVARs. Compared with ZFEN and chimney/snorkel EVARs, PMEGs were used to treat more extensive aneurysms, involving more visceral and renal vessels. However, PMEGs had the lowest unadjusted perioperative death rate (2.7%) compared to ZFEN (3.4%) and chimney/snorkel (6.1%). Stroke rates after PMEG (0.9%) and ZFEN (0.8%) were similar, while chimney/snorkel EVARs had significantly higher stroke rates (3.3%; *p* = 0.03). This study reflects that PMEGs play a significant role in the real-world practice of complex EVARs in the USA, and the short-term results achieved with PMEGs are similar to the benchmark represented by ZFEN and superior to the CE-approved snorkel/chimney EVARs. 

Han and collaborators have reported the technical success of 20 consecutive patients with pararenal AAAs and TAAAs urgently treated with PMEGs. In their hands, technical success was achieved in all patients. A total of 76 renal-mesenteric arteries were treated. There was no 30-day mortality, but major adverse events occurred in 10 patients, consisting of acute kidney injury, blood loss > 1 L, respiratory failure, paraplegia, and ischemic colitis [25]. Subsequently, they reported results on a series of 117 consecutive patients who received PMEGs for complex AAAs or TAAAs. Among those patients, 439 visceral and renal arteries were treated with a 90% procedural technical success. Failure to meet technical success was observed in 11 patients and was most commonly due to the inability to place a covered stent into the target vessel. Six deaths were recorded at 30 days, and eight patients had spinal cord ischemia, with two permanent effects. Two patients had strokes, both with complete full neurologic recovery during follow-up. Acute kidney injury was seen in 13% of the patients. The overall estimated survival rate was 88% at 1 year [9]. 

More recently, in a systematic review and meta-analysis, Gouveia e Melo and colleagues [26] identified 20 studies, including 909 patients treated with PMEGs, 222 of whom had extent I, II, and III TAAAs, while 645 had extent IV TAAAs and pararenal AAAs. Five hundred patients (63.9%) were treated electively, and 282 (36.1%) were treated urgently. The overall technical success was high, at 97.2%, with an overall 30-day mortality rate of 4.4% and major adverse events seen in 15.5% of patients. Although major adverse events were higher at 24.6% in urgent cases compared with 11.6% in elective cases, high technical success was seen regardless of the aneurysm extent or urgency of repair. During follow-up, the overall target vessel patency was 98.9%. Reintervention was required in 19.1% of patients who underwent urgent repairs compared with 8.7% of elective patients, resulting in an overall reintervention rate of 12.3%. 

Of course, the available data present several limitations. In addition to the evident variation in the implementation of PMEGs among different authors, the treated pathologies themselves are diverse. TAAAs, JAAAs, and complex AAAs each have distinct technical requirements, complexities, and outcomes [5]. Furthermore, the mix of elective and urgent cases makes it difficult to accurately analyze the results, and the currently available follow-up duration is severely limited. The results from the studies on physician-modified endografts are considered reliable to a certain extent, but they are limited by the low quality of available data and inconsistent reporting across the studies. The majority of studies were retrospective and single-centered, with limited follow-up, affecting the assessment of the long-term durability of the technique. Additionally, there was a significant lack of homogeneous reporting on outcomes and complications, which could introduce biases and affect the validity of the findings. In addition, a positive publication bias, making available only promising experiences, should be considered in analyzing the presented results. Moreover, regarding the different success rates for emergency and elective surgeries, it should be considered that elective patients are better prepared for the procedure, and there is more time to plan and execute the surgery under optimal conditions. In contrast, urgent cases often involve more complex and extensive repairs, longer operation times, and higher rates of complications due to the emergent nature of the procedure, which can lead to worse outcomes. Lastly, it should be pointed out that the lack of long-term results significantly affects the ability to make sound recommendations on the use of PMEGs. Without adequate long-term data, it is difficult to assess the durability and effectiveness of PMEGs over time, which are critical factors in determining overall benefits and risks. Consequently, the recommendations outlined in the remainder of this article are intended as provisional, based primarily on short-term results.

## 4. Medico-Legal Implications

Regarding the regulatory framework, the use of PMEGs falls within the off-label use of medical devices, regulated in Europe by Regulation (EU) 2017/745 [27] (Medical Devices Regulation—MDR) and in the US by the Food and Drug Administration (FDA) Modernization Act of 1997 [28]. 

Regulation (EU) 2017/745 does not prohibit the off-label use of medical devices such as modified vascular prostheses; however, such use must be justified by healthcare professionals based on clinical evidence and patient needs when suitable on-label alternatives are not available. The regulation primarily regulates the marketing and distribution of medical devices, ensuring that they meet stringent safety and performance requirements prior to approval. Once a device is on the market, healthcare professionals are required to assess on a clinical basis the appropriateness of using these devices off-label, although this practice must be supported by existing clinical evidence, or a documented rationale based on the principles of medical necessity and informed consent. 

Even the Food and Drug Administration Modernization Act of 1997 does not prohibit the off-label use of medical devices. US regulations allow healthcare professionals to use medical devices off-label when they believe it is in the best interest of the patient. As in the EU, this use must be well supported by clinical judgment and evidence when possible. However, manufacturers may not market their devices for off-label use. This restriction aims to ensure that the promotion of devices remains within the FDA-approved indications. Protocols for off-label use are not imposed by the FDA, but are often governed by professional standards and liability considerations within the medical community. 

Both the European Union and the United States, therefore, do not explicitly codify protocols for off-label use of medical devices such as modified vascular prostheses, leaving considerable discretion to medical professionals. This practice emphasizes the balance between regulatory oversight and physician autonomy in making patient-centered decisions. The absence of specific, codified protocols for off-label use means that such actions are primarily governed by the clinical judgement of healthcare professionals, who must weigh the evidence and need for such use against the potential risks and benefits to their patients. While this allows some flexibility in clinical practice, it also imposes considerable responsibility on healthcare professionals to ensure that their decisions are well founded on evidence and best practice, reflecting both regulatory standards and patient safety concerns. 

Having provided this introduction, the discussion now turns to a brief examination of the main medico-legal implications associated with the clinical use of PMEGs, of which there are essentially three: standardization and safety, medical liability, product liability, and informed consent.

### 4.1. Standardization and Safety

Several authors reported numerous techniques for creating PMEGs, but there are no universally accepted protocols. Variations in methodologies, such as the use of different basic devices, reinforcing materials and surgical instruments, can significantly influence the results of the procedure. This raises obvious questions about the consistency and repeatability of the results obtained with PMEGs, since without a common protocol it becomes complicated to compare success rates, complications, and relapses between different experiences.

Addressing the issue of standardizing procedures for creating PMEGs becomes therefore essential to ensure safe and reliable outcomes for patients. Standardized protocols defining the requirements for the creation, implementation and monitoring of PMEGs are needed. These protocols should be evidence-based and regularly updated to reflect technological and clinical developments in the field of vascular surgery.

To date, the only attempt at technical standardization of a PMEG approach has been made by an Italian research group [8], which defined a PMEG mounting method for complex abdominal aortic aneurysms based on specific anatomical selection criteria, measurement method, and standard modification technique.

### 4.2. Medical Liability

The creation of PMEGs requires an in-depth knowledge of vascular anatomy and anatomical relationships, as well as an understanding of how the available endografts work. The variety of anatomical abnormalities and pathological conditions makes each procedure unique, requiring the surgeon to adapt the technique in a flexible and sometimes creative manner [29]. After all, the ‘individuality’ of each patient is an inherent characteristic of all areas of surgery, and of vascular surgery in particular [30], as stated by the European Society for Vascular Surgery, which, in its guidelines on the management of abdominal aorto-iliac artery aneurysms, affirms *“… “under no circumstance should […] be seen as the legal standard of care in all patients […] the document provides a guiding principle, but the care given to an individual patient is always dependent on many factors including symptoms, comorbidities, age, level of activity, treatment setting, available techniques and other factors”* [31].

Clearly, as PMEGs are customized for each patient, there is no one-size-fits-all approach. Surgeons must be able to accurately assess the patient’s anatomy, plan the procedure in detail, and adapt the technique of creating PMEGs to the specific needs of the case. This requires problem solving skills and flexibility during surgery. The manipulation of endografts during the creation of PMEGs requires extreme precision and extraordinary fine hand coordination. Surgeons must be able to use delicate surgical instruments with millimetric precision, ensuring the correct positioning of fenestrations and thus adaptability to the patient’s anatomy. In addition, the ability to manage any intraoperative complications requires great experience and calmness under pressure.

Hence, it is clear that such technical criticality profiles must be duly and carefully taken into account in the event of adverse events abstractly attributable to malpractice profiles. Each country provides for specific and particular ways of framing medical malpractice, both under civil and criminal law, but generally, the technical difficulty of the procedure is one of the parameters that is considered in assessing the doctor’s fault, together with the existence (and therefore the observance) of specific operating protocols codified and validated by the scientific community. The creation and implantation of PMEGs are not only technically very demanding, but also lack unambiguous references in terms of operating methods (standardization), also in view of the absolute uniqueness of each patient, and therefore of each case. The combination of these two features makes it very likely that, in the event of an ascertained malpractice, the degree of culpability would be tempered by the fact that the error, if ascertained, was committed not in the context of a routine or otherwise well-codified intervention, but in the context of an intervention of very high technical complexity, customized (and therefore unique) and in the absence of codified procedural guidelines.

### 4.3. Product Liability

When a physician decides to modify an endograft, the original product, approved by regulatory agencies, is altered in ways that may affect both its functionality and safety. In these cases, the question of the manufacturer’s liability for product defects becomes problematic [32]. 

From a legal point of view, the manufacturer could argue that it is not liable for defects or failures of the endograft that are a consequence of modifications made by the physician, since these modifications are not part of the intended and approved use of the device. Indeed, modifications made by the physician could exclude the manufacturer from traditional liability for manufacturing defects, design, or lack of appropriate warnings, unless it can be shown that the defect pre-existed the modification.

On the other hand, patients harmed by a PMEG might have grounds to pursue legal action against both the manufacturer and the doctor, depending on the specific circumstances of the case, such as the manufacturer’s instructions regarding the modifiability of the device and the doctor’s compliance with the standards of good medical practice. In addition, the hospital or healthcare institution could be involved in legal action if it is shown that they did not provide adequate supervision or protocols in the use of PMEGs.

In principle, when the surgeon modifies the device and uses it off-label, the manufacturer is exempt from any product liability claims. This is why it is absolutely advisable for surgeons to pursue a formal investigational device exemption (IDE). Investigational device exemption allows the use of unapproved medical devices in clinical research to collect safety and efficacy data necessary for product development [33]. In practical terms, obtaining an IDE involves several steps. First, the physician or sponsoring institution must submit an IDE application to the responsible regulatory agency (EMA in Europe and FDA in the US), providing detailed information about the device, the intended modifications, and the clinical study protocol. This application must include comprehensive data on the device’s design, manufacturing processes, and preclinical testing results, demonstrating the device’s safety and potential effectiveness. The clinical study protocol should outline the study design, objectives, patient selection criteria, and methods for data collection and analysis. Additionally, the application must include informed consent documents, investigator qualifications, and a risk analysis. Once the regulatory agency reviews and approves the IDE application, the clinical study can commence, during which the modified device is used on patients under strict regulatory oversight to collect safety and efficacy data. Throughout the study, regular progress reports must be submitted to the regulatory agency, detailing any adverse events, protocol deviations, and interim findings.

With particular reference to the European regulatory context, it should be noted that Regulation (EU) 2017/745 explicitly provides in Article 61 (paragraphs 4–6) for cases in which class III devices (high-risk devices, such as most implantable devices, those containing drugs or animal derivatives, and certain devices that interact with the functions of vital organs) must be adopted in the context of clinical studies. It is particularly useful to quote the text of the Regulation:

*“4. In the case of implantable devices and class III devices, clinical investigations shall be performed, except if: *

- *the device has been designed by modifications of a device already marketed by the same manufacturer,*
- *the modified device has been demonstrated by the manufacturer to be equivalent to the marketed device, in accordance with Section 3 of Annex XIV and this demonstration has been endorsed by the notified body, and*
- *the clinical evaluation of the marketed device is sufficient to demonstrate conformity of the modified device with the relevant safety and performance requirements.*

*In this case, the notified body shall check that the PMCF plan is appropriate and includes post market studies to demonstrate the safety and performance of the device. In addition, clinical investigations need not be performed in the cases referred to in paragraph 6.*

*5. A manufacturer of a device demonstrated to be equivalent to an already marketed device not manufactured by him, may also rely on paragraph 4 in order not to perform a clinical investigation provided that the following conditions are fulfilled in addition to what is required in that paragraph:*

- *the two manufacturers have a contract in place that explicitly allows the manufacturer of the second device full access to the technical documentation on an ongoing basis, and*
- *the original clinical evaluation has been performed in compliance with the requirements of this Regulation, and the manufacturer of the second device provides clear evidence thereof to the notified body.*

*6. The requirement to perform clinical investigations pursuant to paragraph 4 shall not apply to implantable devices and class III devices:*

*(a)*  *which have been lawfully placed on the market or put into service in accordance with Directive 90/385/EEC or Directive 93/42/EEC and for which the clinical evaluation:*
  - *is based on sufficient clinical data, and*
  - *is in compliance with the relevant product-specific CS for the clinical evaluation of that kind of device, where such a CS is available; or*
*(b)*  *that are sutures, staples, dental fillings, dental braces, tooth crowns, screws, wedges, plates, wires, pins, clips or connectors for which the clinical evaluation is based on sufficient clinical data and is in compliance with the relevant product-specific CS, where such a CS is available”*

In summary, for PMEGs, the approval process can be simplified if the modified device is based on an existing and approved model, demonstrating that the modifications do not compromise the safety and efficacy of the device. This allows surgeons to use modified versions of existing devices without the need for extensive clinical studies, provided that all conditions of equivalence and documentation are met and approved by a notified body.

### 4.4. Informed Consent

Since PMEGs involve the personalized modification of medical devices and present specific risks, it is essential that patients fully understand the available treatment options, the associated potential risks and benefits, and the possible alternatives [34,35]. In particular, it is crucial to clearly explain three key aspects to patients: the possible alternative treatment strategies (open surgical treatment or classical endovascular approaches), the absence of codified protocols establishing how endograft modification should be performed, the risks and possible complications associated with the procedure, and the particular technical complexity of the procedure.

From a medico-legal point of view, the last aspect is particularly interesting. Surgeons have an ethical duty to acknowledge their professional limitations and to be transparent about their experience and expertise in the specific field of the proposed procedure [29,30,31,32,33,34,35,36]. This means that if the surgeon proposing the procedure does not have the necessary training or experience to perform the procedure safely and effectively, he has an ethical duty to clearly communicate this to the patient and refer him to a more qualified colleague.

Furthermore, the informed consent process should include a detailed discussion of how PMEG is tailored to the patient’s specific anatomical and clinical needs, highlighting the personalized nature of the procedure, which may lead to variable and not always predictable outcomes. It should also address the lack of long-term data on the duration and performance of PMEGs, which may be an important consideration for patient decision-making [37]. In addition, the discussion should include an overview of post-operative expectations [38], including monitoring and potential follow-up procedures.

This comprehensive approach ensures that patients are not only aware of the immediate surgical risks, but also understand the long-term commitment and follow-up required by these personalized treatments. Ultimately, by facilitating a more informed and shared decision-making process, patients can feel more confident and secure in their choices, thus improving both patient satisfaction and treatment outcomes.

In practical terms, given the type of intervention and its customized nature, it is necessary for the form to be collected in written form, in order to formally document the provision of complete and exhaustive information, and above all, consistent with the patient’s individual characteristics. The need for written consent in the context of clinical trials is moreover established at European level by Directive 2001/20/EC of the European Parliament [39].

## 5. Conclusions

Physician-modified endoprostheses (PMEGs) represent a major therapeutic option in the treatment of complex aortic aneurysms that cannot be corrected by traditional surgical techniques.

As emerged at the outcome of the proposed analysis, however, there are concrete obstacles to an extensive application of this innovative therapeutic strategy, which is both surgical and medico-legal in nature. Beyond the particular technical complexity, requiring important surgical skills and not inconsiderable operating experience, there is the primary medico-legal problem of standardization, which is complex to resolve given the “customised”, i.e., “patient-tailored” nature of prostheses. As widely discussed, the European and US regulations on medical devices do not prohibit the possibility of off-label use of medical devices such as vascular prostheses, but it is, of course, desirable that strict protocols, vetted by the scientific community, regulate and govern such use.

It is crucial that the medical community and regulators work together to formulate clear guidelines and standard protocols for the use of PMEGs. These efforts will help ensure that modifications to endoprostheses are performed safely, further improving patient safety and minimizing the risk of medical litigation. Only through collaboration, continuing education, and research can we effectively address the complex medico-legal issues presented by the innovative use of PMEGs in vascular surgery.

Ultimately, as pointed out in the first section of the article, it must be considered how the reliability of the literature data examined and used as the basis for medical-legal considerations are necessarily characterized by limitations, including variation in the implementation of PMEGs among different authors, the diversity in treated pathologies (TAAA, JAAA, complex AAA), each with distinct technical requirements and outcomes, the inclusion of both elective and urgent cases, limited follow-up duration, and potential positive publication bias. Consequently, long-term follow-up studies will be necessary in the future in order to come to more complete and comprehensive conclusions and recommendations.

## Figures and Tables

**Table 1:** Main differences between traditional and physician-modified endovascular prostheses.

	EVAR (Standard Endovascular Aortic Repair)	PMEGs (Physician-Modified Endografts)
Definition	Prefabricated aortic prostheses used for the endovascular treatment of abdominal aortic aneurysms	Aortic prostheses modified by doctors to suit the specific needs of the patient
Customization	Limited to predefined sizes and configurations	High, customized to the patient’s anatomy
Production times	Immediate availability as prefabricated	More time needed for modification and customization
Indications for use	Suitable for patients with anatomies conforming to device specifications	Used in patients with complex anatomies
Success rates	High in suitable anatomies	Variable, but often the only option for complex anatomies
Risks	Minimal, standardized and well documented	Potentially higher due to modifications, but advantageous in complex situations
Surgical approach	Standardized, with well-defined protocols	Individualized according to the needs of the patient and the changes made
Intervention duration	Generally shorter due to standardization	Potentially longer due to modifications and preparation
Follow-up and maintenance	Regular and standardized according to established guidelines	Customized and potentially more intensive due to modifications
Supporting clinical evidence	Widely supported by clinical studies and long-term data	Limited evidence based mainly on clinical cases and fewer studies

## Data Availability

The original contributions presented in this study are included in the article, and further inquiries can be directed to the corresponding author.
